# Introducing *Wearable Technologies*: An open access journal focused on the design, control and mechanics of wearable devices

**DOI:** 10.1017/wtc.2020.2

**Published:** 2020-09-04

**Authors:** Sunil K. Agrawal

**Affiliations:** Department of Mechanical Engineering and Rehabilitation Medicine, Columbia University, New York, NY USA

It is my pleasure to welcome you to *Wearable Technologies*, a new Open Access journal devoted to the science, technology, and applications of wearable robotics. While many of us routinely count our daily steps using smart watches and navigate our cars through traffic using cell phones, wearable robotics has the potential to take this technology one step further in its ability to impact our daily lives. For instance, a wearable exoskeleton can empower an industrial worker to complete his or her daily activities more efficiently while minimizing excessive fatigue. Through the application of a different human-machine interface on a similar exoskeletal device, one has the potential to adapt this control framework to serve an alternative purpose, such as to empower a stroke survivor to relearn walking and improve coordination.

Wearable technologies have a widespread role across areas including rehabilitation, construction, environment exploration, injury prevention, diagnostics, sports, fitness, and leisure activities. Key elements of wearable technologies include sensing, computation, actuation, communication, and wearability. Robotics researchers around the world critically study these elements, some analyzing them individually, others combining them in powerful ways, all working to complement and enhance human ability. With growing academic and commercial focus on enhancing productivity and human ability, new scientific methods and interventions are being developed across diverse fields, all ultimately with the shared goal of improving human health and well-being. In healthcare, applications of wearable devices include clinically improving human functions and obtaining better measurements of human physiology to strengthen clinical evidence and decision making. Artificial intelligence tools are also being used to assist with real-time decision making as well as their robotic implementations with embedded actuators.

The launch of this journal will create a new forum for researchers to collaborate and share ideas centered around this actively developing area of wearable technologies. The launch also carries a responsibility for all of us to critically evaluate the field and its translation to and impact on the society at large. The focus of this journal will be on the design, control and mechanics of wearable technologies. Topics will include, but are not limited to exoskeletons, exosuits, prosthetics, intelligent orthotics, soft wearable systems, wearable sensors, wearable monitors, human-robot interactive controllers, and virtual reality wearable devices.

The editorial board for Wearable Technologies includes prominent researchers from across the world. I want to take this opportunity to sincerely thank our editorial board members from Asia (Prof. G. K. Anathasuresh, Prof. Q. Wang, and Prof. P. Schull), from Europe (Prof. D. Farina, Prof. D. Floreano, Prof. N. Vitiello), and from USA (Prof. P. Artemiadis, Prof. H. Huang, Prof. J. Pons, Prof. J. Rosen, Prof. T. Sugar, and Prof. D. Zanotto). I also want to acknowledge the dedication of Ms. E. Heppenstall and her colleagues at Cambridge University Press for bringing this journal to our robotics community.

## Data Availability

Data availability is not applicable to this article as no new data were created or analysed in this study.

